# Palaeohistology and life history evolution in cave bears, *Ursus spelaeus* sensu lato

**DOI:** 10.1371/journal.pone.0206791

**Published:** 2018-11-21

**Authors:** Kristof Veitschegger, Christian Kolb, Eli Amson, Torsten M. Scheyer, Marcelo R. Sánchez-Villagra

**Affiliations:** 1 Paleontological Institute and Museum, University of Zurich, Zurich, Switzerland; 2 Naturalis Biodiversity Center, Leiden, The Netherlands; 3 AG Morphologie und Formengeschichte, Bild Wissen Gestaltung—ein Interdisziplinäres Labor and Institut für Biologie, Humboldt-Universität, Berlin, Germany; 4 Bild Wissen Gestaltung, Ein interdisziplinäres Labor, Humboldt Universität, Berlin, Germany; 5 Museum für Naturkunde, Leibniz-Institut für Evolutions- und Biodiversitätsforschung, Berlin, Germany; Liverpool John Moores University, UNITED KINGDOM

## Abstract

The abundance of skeletal remains of cave bears in Pleistocene deposits can offer crucial information on the biology and life history of this megafaunal element. The histological study of 62 femora from 23 different European localities and comparisons with specimens of five extant ursid species revealed novel data on tissue types and growth patterns. Cave bear’s femoral bone microstructure is characterized by a fibrolamellar complex with increasing amounts of parallel-fibered and lamellar bone towards the outer cortex. Remodelling of the primary bone tissue initially occurs close to the perimedullary margin of the bone cortex around the linea aspera. Although similar histological traits can be observed in many extant bear species, the composition of the fibrolamellar complex can vary greatly. Cave bears reached skeletal maturity between the ages of 10 and 14, which is late compared to other bear species. There is a significant correlation between altitude and growth, which reflects the different body sizes of cave bears from different altitudes.

## Introduction

During the Pleistocene, Eurasia was home to many large sized mammals, commonly referred to as megafauna [1, 2]. Among them are cave bears, *Ursus spelaeus*, one of the most commonly found species, recovered in numerous Eurasian localities ranging from Spain to Russia [3, 4]. The closest living relatives of *U*. *spelaeus* are the brown bear, *U*. *arctos* and the polar bear, *U*. *maritimus* [5, 6]. The lineage of cave bears split between 2.75 and 1.4 Ma [5, 6] from the aforementioned two taxa. The hypothesized ancestral species of cave bears, *U*. *deningeri* is according to some authors, directly on the evolutionary line of *U*. *spelaeus* and, thus, the anagenetic ancestor of the latter [3, 7]. Cave bears occupied a wide range of habitats and remains of this species were found from about sea level up to 3000 m in altitude [8]. Molecular studies have shown that several distinct haplotype groups of cave bears are recognisable [9–12]. Some authors suggest giving species and subspecies status to some of those [8, 13] but it remains unclear if species status can be assigned to those cave bear haplotypes [4]. As this discussion is not completely resolved yet, we use *U*. *spelaeus* sensu lato, including *U*. *ingressus*, *U*. *s*. *spelaeus*, *U*. *s*. *eremus*, and *U*. *s*. *ladinicus* in the text but include analyses considering these haplotypes and possible species. High altitude specimens of *U*. *spelaeus* s.l. are often smaller [8]. High altitudes, in general, represent a challenging environment for mammals due to hypoxic conditions, which impair growth [14, 15].

Bone histology of extinct mammals has received increased interest in recent years, as details found in thin-sections provide insights into growth and thus life history [16–21]. These works focus on deposition of different bone tissue types and/or by analyzing growth cycles by measuring the distance between lines of arrested growth (LAGs) or growth annuli [22]. Cave bears went into dormancy during winters and therefore spent a considerable amount of time inactive during the year [23]. Non-hibernating mammals risk losing balance of bone resorption and formation in favour of resorption when being inactive for extended periods, which leads to extensive loss of bone minerals in a short time [24]. Studies on extant black bears, *U*. *americanus*, however, show that the balance between resorption and formation is not affected by extended inactive periods during winters but the cortical bone turnover rate is lower [25, 26]. The cortical bone geometry and strength of the femoral midshaft remain unchanged [26].

Much of our work is based on previous knowledge of bone formation and change, including Amprino’s rule, which postulates that different bone tissue types are produced in variable rates [27]. The fastest bone tissue to be produced is woven-fibered bone, followed by parallel-fibered and lamellar bone [28, 29]. Besides these three, a fibrolamellar complex is also found in mammals. During the formation of this complex initially highly vascularised woven-fibered scaffolding is deposited. In a later stage, the mostly reticular or plexiform vascular canals are filled with parallel-fibered and lamellar bone [29]. Recently, the term fibrolamellar complex has come under scrutiny as it does not provide detailed information about formation or structure of primary bone tissue [30]. Nonetheless, we continue using fibrolamellar complex to ensure comparability to previous studies [21]. A LAG is a thin opaque or translucent, circumferential line of compact bone and indicates a cessation of growth [22, 29]. In recent years, evidence accumulated supporting the yearly formation of LAGs and that this pattern is independent from climate or metabolic rate [18, 29, 31–34]. Here, we aim to study several aspects of the bone histology of cave bears: (1) we describe the general histology of the femora of cave bears compared to extant relatives, (2) we aim to reconstruct growth of cave bears by using average inter-LAG distance (AILD) as a proxy and compare it to extant relatives, (3) we present a hypothesis on some life history variables for cave bears based on the investigated growth, and (4) we examine the intraspecific variation in growth in the context of the altitude of the excavation site in order to elucidate if the investigated growth is different in the smaller cave bear populations from high altitudes.

## Material and methods

No permits were required for the described study, which complied with all relevant regulations. All sampled specimens are part of museum collections. The providing institutions and curators/collection managers are named in the material and methods section and acknowledgments.

**Fig 1:**
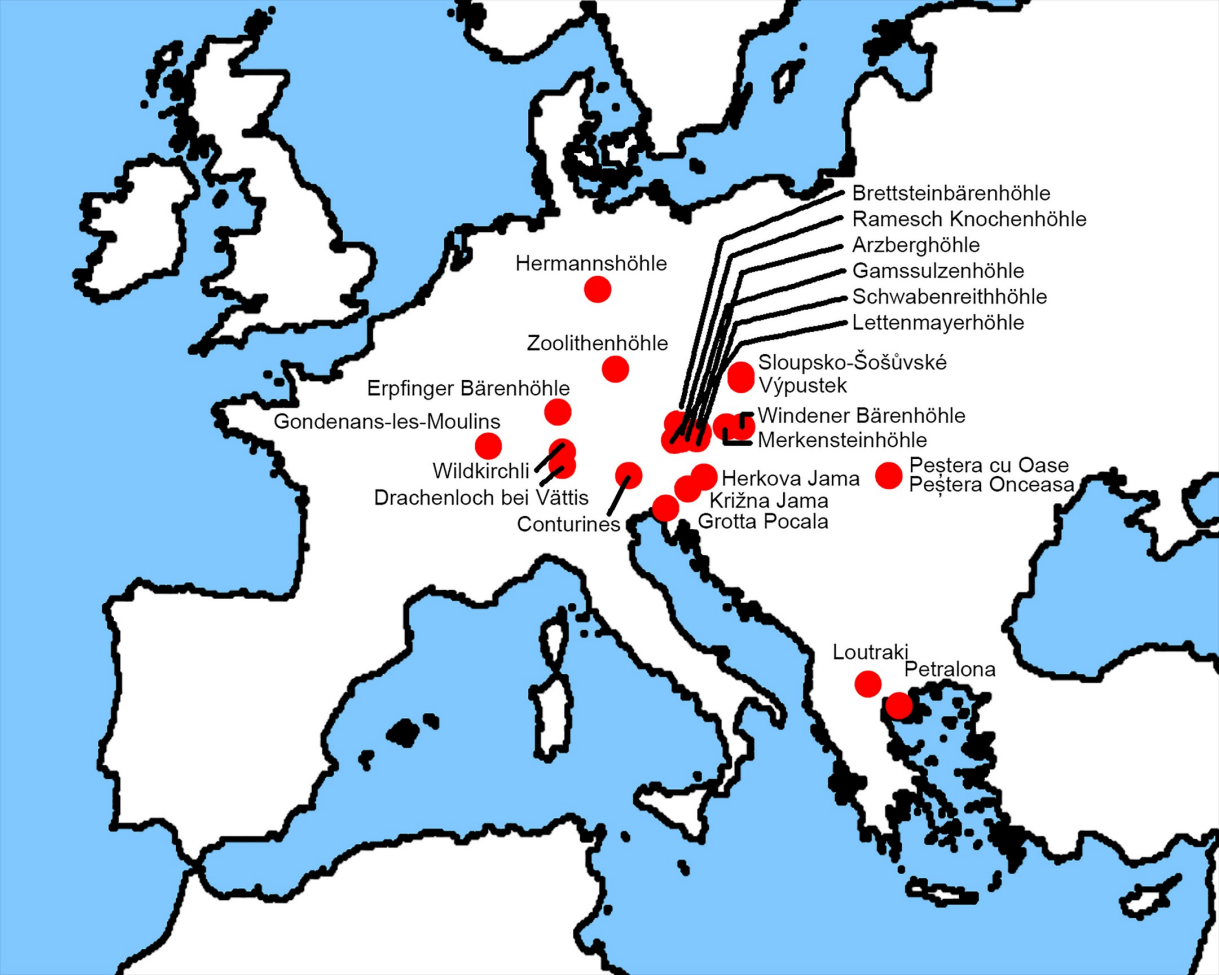
Map of sampled locations.

We investigated 62 femora of cave bears (*U*. *spelaeus* s.l.) from 23 different localities (Fig 1, S1 Table), as well as specimens of one other extinct (*U*. *deningeri*) and five extant species of Ursidae. *U*. *deningeri* is represented by three femora. From the 23 localities, twelve preserved the haplotype *U*. *ingressus* and seven *U*. *spelaeus* s.s. For the remaining four it remains unclear which haplotype was preserved at their respective caves (S2 Table).

The following institutions provided material for sampling from their collections: Aristotle University of Thessaloniki—School of Geology (AUTH); Biologiezentrum Linz (BZL); Canadian Museum of Nature (CMN); Finnish Museum of Natural History (MZH); Institut für Paläontologie, Universität Erlangen (IPUE); Institut für Paläontologie, Universität Wien (PIUW); Museum für Naturkunde Berlin (MfN); Muséum national d'Histoire naturelle, Paris (MNHN); Naturhistorisches Museum Basel (NMB); Naturhistorisches Museum Wien (NHM); Naturmuseum Südtirol Bozen (PZO); Naturmuseum St. Gallen (NMSG); Paläontologische Sammlung der Universität Tübingen (GPIT); and Swedish Museum of Natural History (SMNH). The thin-sections prepared and investigated for this study are deposited in the corresponding collections with the inventory number of their femora (S1 Table).

The sampled femora of extant bear species were those of *Helarctos malayanus* (one), *Melursus ursinus* (one), *U*. *americanus* (two), *U*. *arctos* (six), and *U*. *maritimus* (two), For *U*. *spelaeus* s.l. and *U*. *arctos*, ontogenetic series were available and in case of *U*. *arctos* the approximate age of three individuals was known (S1 Table). The preparation of the thin-sections followed standard procedures [17, 20, 35]. The midshaft of the femur was chosen, as it preserves the most complete growth record [17, 36]. We investigated bone growth in accordance to Kolb et al. [17] by measuring the distance between consecutive LAGs (S1 Table). Thus, more precisely, we investigated the amount of bone deposited in a growth cycle as proxy for growth in the respective cycle. To differentiate this form of growth studies [17, 20] from studies more concerned with average growth rate [37], we refered to our proxy as “average inter-LAG distance” (AILD) The histology of the bones was observed under normal transmitted or cross-polarized light using a Leica DM 2500 M composite microscope. In many cases, histological details were more pronounced using a lambda compensator. Pictures were taken using a Leica DFC 420 C digital camera. Growth zones were defined as the distance between two LAGs and measured using Leica IM 50 Image Manager software.

**Fig 2:**
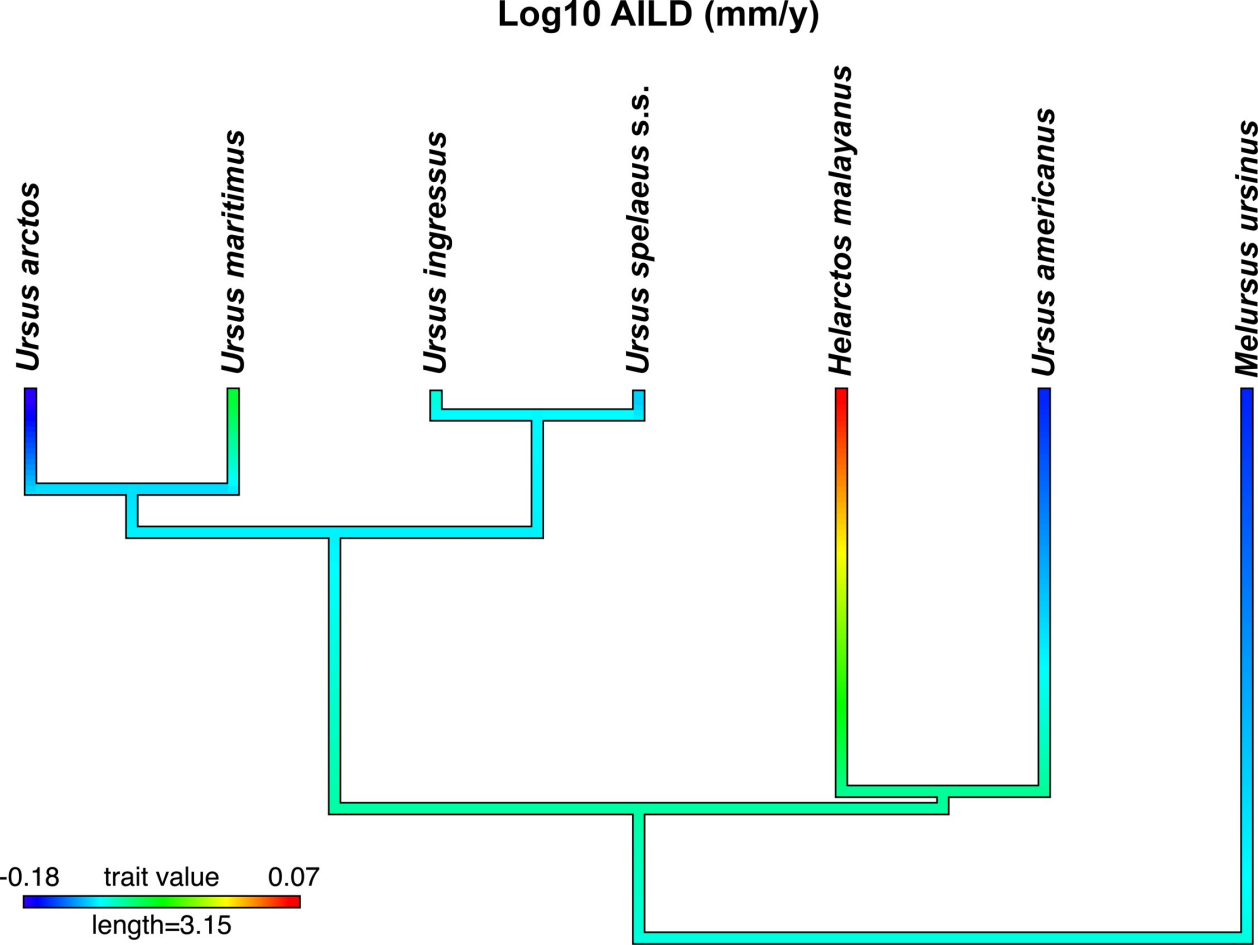
Phylogenetic framework for this study. Log10-transformed AILD was mapped on the time tree using a gradient from blue (low values) to red (high values).

Measurements were acquired in the medial quadrant of the thin-sections of the femora because in this area LAGs were least obscured by remodelling (Figures A, B, I in S1 File). Pre-OCL inter-LAG distance was averaged (AILD) to compare it among species (S2 Table). The outer circumferential layer (OCL) denotes skeletal maturity [29]. Thus, we avoided bias resulting from mixing pre-maturity AILD with post-maturity AILD. For the comparison of AILD among different cave bear localities, growth zones 2 to 7 were averaged to include also samples lacking a full growth record (S2 Table). These averaged variables were also used as basis to compare the slopes and intercepts between *U*. *spelaeus* s.s. (sensu stricto) (incl. *U*. *s*. *eremus* and *U*. *s*. *ladinicus*) and *U*. *ingressus*. Comparative methods were employed to identify and eventually take into account the phylogenetic relationships of the sampled taxa. We investigated the species/haplotype averaged data with a PGLS (phylogenetic generalized least squares) as implemented in the R packages ape [38] and caper [39] using maximum likelihood (see also Losos [40]). We used a model defined by a variance-covariance matrix that reflects the phylogenetic signal observed in the data [41, 42]. It retrieves the phylogenetic signal index lambda (λ; [43]), of which a value of 0 reflects that data are independent from phylogeny (all nodes of the tree are collapsed resulting in a star phylogeny data structur), a value of 1 denoting that data distribution is consistent with a Brownian motion model of evolution (BM; no nodes are collapsed; [43]). We retrieved the phylogenetic information from Bon et al. [5], a tree based on mitochondrial genome and that included all sampled species. Knapp et al. [4] was used to place *U*. *ingressus* in the phylogeny. Branch lengths representing time of divergence among our investigated taxa were retrieved from Nyakatura and Bininda-Emonds [44], Bon et al. [5], and Knapp et al. [4]. The analyses excluded *U*. *deningeri* because of the position of this species in the cave bear phylogeny [3, 7]. AILD was mapped onto the built timetree (function contMap, package phytools), for which maximum likelihood reconstruction of the ancestral states is performed [45]. The phylogenetic framework is shown in Fig 2.

We investigated the possible relationship between AILD and the altitude of different localities. For this, we used OLS regressions and Kendall’s tau to statistically assess the correlation of the variables. In this study, the greatest latero-medial diameter (LMD) of the midshaft measured was used as proxy for body size for a species or locality and compared to AILD. Altitude was compared to AILD and midshaft LMD. To further study if the effect of altitude was size-dependent, we used size-corrected AILD by investigating the residuals of an OLS regression of the AILD against midshaft LMD and compared these residuals with altitude. AILD and midshaft LMD were log10-transformed. Statistical analyses were performed in R, version 3.2.3 [46] and with the additional package Kendall [47]. Plots were produced with ggplot2 [48] and Adobe Illustrator CS5.

## Results

### Skeletochronology

**Fig 3:**
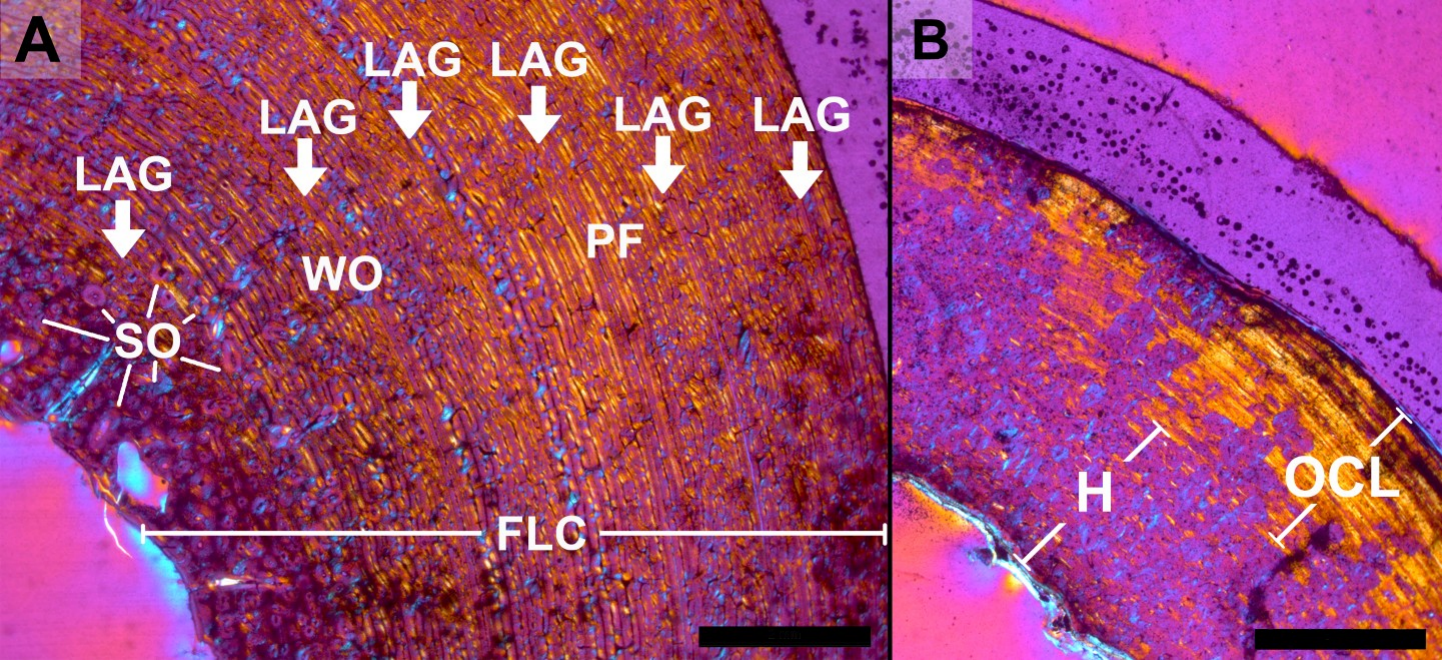
**Overview of histology of bears exemplified with *Ursus spelaeus* s.l. MB. Ma. 10881 (A) and *Ursus arctos* MNHN 1904–244 (B).** The thin-sections of the medial part of the femoral midshaft are shown in cross-polarised light with lambda compensator. White arrows indicate LAGs, H = Haversian tissue, FLC = fibrolamellar complex, PF = parallel-fibered bone (mainly), SO = secondary osteon, WO = woven-fibered bone (mainly). (Scale bars: 2 mm).

An overview of the histological features identified in this study is depicted on Fig 3.

**Fig 4:**
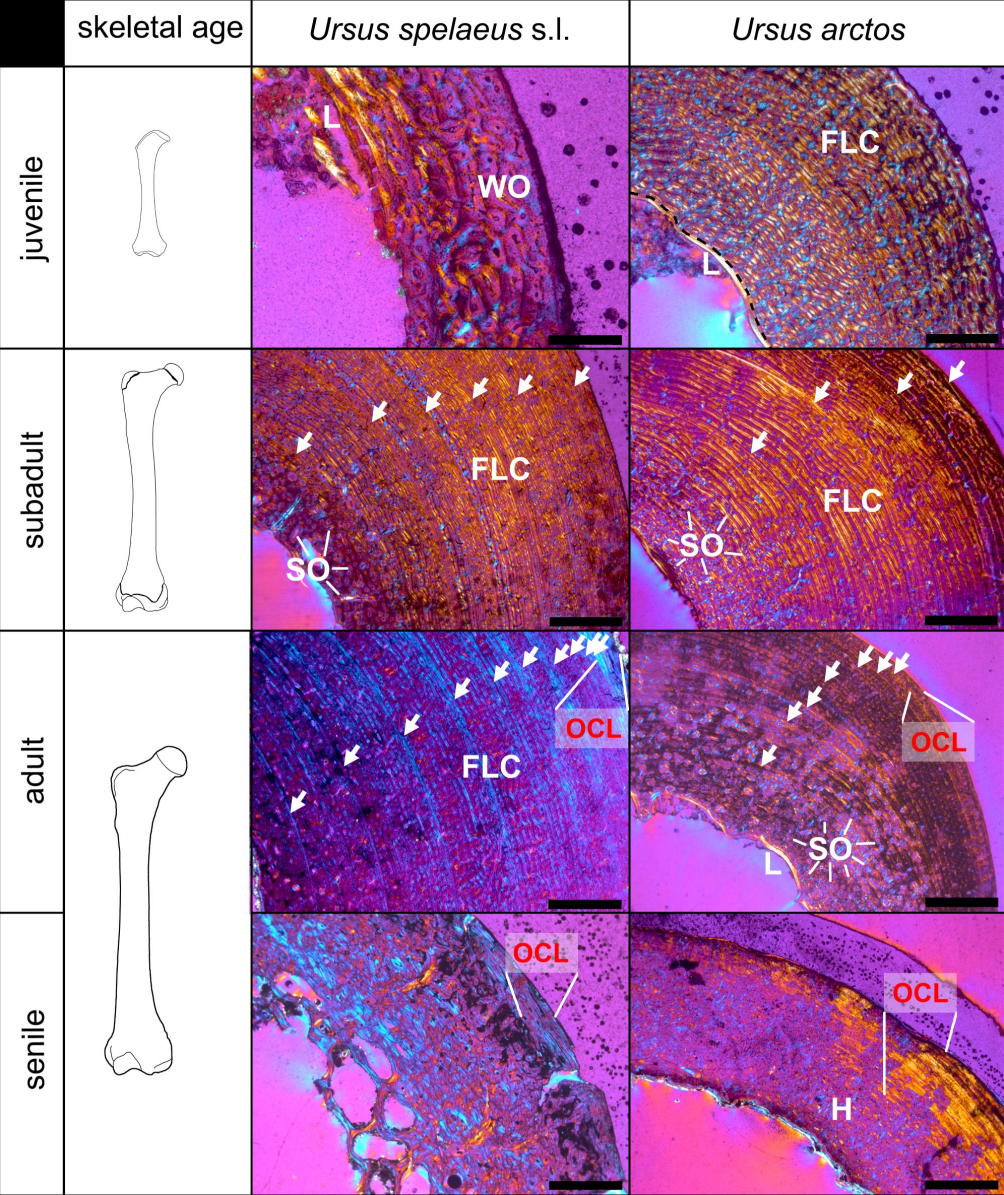
Ontogenetic change of bone histology in the medial part of the femur midshaft of *Ursus spelaeus* s.l. and *Ursus arctos* (pictures under cross-polarized light with lambda compensator). White arrows indicate LAGs. Juvenile *Ursus spelaeus* s.l. PZO 5136 (Scale bar: 0.5 mm), subadult *Ursus spelaeus* s.l. MB.Ma.10881 (MfN) (Scale bar: 2 mm), adult *Ursus spelaeus* s.l. PEC 1183 (AUTH) (Scale bar: 2 mm), senile *Ursus spelaeus* s.l. LAC 6277b (AUTH) (Scale bar: 2 mm), juvenile *Ursus arctos* SMNH 2016–5132 (Scale bar: 1 mm), subadult *Ursus arctos* SMNH 2016–5131 (Scale bar: 2 mm), adult *Ursus arctos* SMNH 2016–5025 (Scale bar: 2 mm), senile *Ursus arctos* MNHN 1904–244 (Scale bar: 2 mm). Note that lamellar bone in the outer cortex of adult and senile specimens is indicated as OCL. H = Haversian tissue, FLC = fibrolamellar complex, L = lamellar bone, SO = secondary osteon, WO = woven-fibered bone (mainly).

Femora of neonate cave bears exhibit a fibrolamellar complex with high amounts of woven-fibered bone tissue. In the posterior part of the cortex, high amounts of parallel-fibered bone are deposited. The vascularization is mostly longitudinal as well as reticular and the parallel-fibered bone located posteriorly is less vascularised. With increasing individual age, the amounts of parallel-fibered and lamellar bone within a fibrolamellar complex increase while vascularization changes to predominantly laminar and plexiform arrangement. In the outer cortex of adult animals, an avascular OCL is present. A narrow lamellar endosteal layer and/or trabecular bone can be found in many individuals. In senile animals, a scarcely vascularised mixture of woven-fibered and parallel-fibered bone is present. The few vascular canals are usually longitudinally arranged (Fig 3). A varying amount of parallel-fibered and lamellar bone is the most frequent histological variation among *U*. *spelaeus* s.l. femora (Figures A, B, I in S1 File). Juvenile *U*. *arctos* exhibits a fibrolamellar complex dominated by woven-fibered bone with plexiform and laminar vascularisation. In the innermost part of the cortex, a thin endosteal layer comprised of avascular, laminar bone can be distinguished. Older animals exhibit a fibrolamellar complex with a higher amount of parallel-fibered bone in the outer parts of the cortex, where the vascularisation is laminar and plexiform. The overall change in histology from the inner to the outer part of the cortex is not very distinct. An endosteal layer and OCL are present (Fig 4, Figure F in S1 File).

In accordance with the already described histology of cave bears and brown bears, the femora of *H*. *malayanus*, *U*. *americanus*, *U*. *deningeri*, and *U*. *maritimus* exhibit a fibrolamellar complex with increasing amounts of parallel-fibered bone towards the outer surface of the cortex. The vascularization is primarily laminar and plexiform. In the outer cortex of the femora of *H*. *malayanus*, *U*. *americanus*, and *U*. *maritimus*, an OCL is present (Figures C, E, G, H in S1 File).

**Fig 5:**
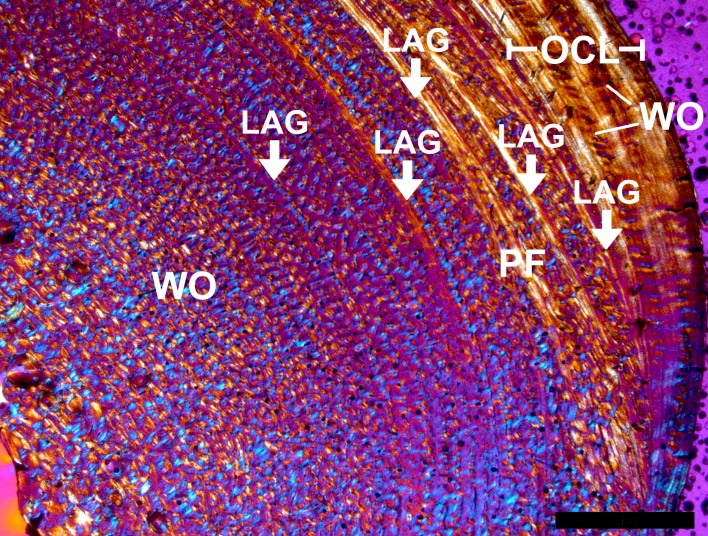
Histology of the medial part of the femoral midshaft of *Melursus ursinus* (MNHN 1879–307). The thin-section is shown in cross-polarised light with lambda compensator. White arrows indicate LAGs, OCL = outer circumferential layer (mostly formed by lamellar bone), PF = parallel-fibered bone, WO = woven-fibered bone. Note the woven-fibered bone within the OCL. (Scale bars: 2 mm).

The bone of *M*. *ursinus* exhibits a very narrow medullary cavity, as also depicted by Mátyás [49]. On the whole, histology in *M*. *ursinus* varies among the anterior, medial, posterior, and lateral quadrants of the femoral cortex. The anterior part of the bone exhibits a matrix of woven-fibered and parallel-fibered bone with mostly longitudinal and reticular vascularization in the inner part of the cortex (fibrolamellar complex). The outer part of the cortex of the anterior quadrant of the bone shows a higher amount of parallel-fibered bone and a distinct avascular OCL. Medially, the inner cortex of the bone is comprised of woven-fibered bone with mostly longitudinal and reticular vascularization whereas the outer cortex exhibits parallel-fibered bone with low amounts of laminar vascularization and layers of avascular lamellar bone. Medially, the lamellar bone of the OCL is interlayered by woven-fibered bone with longitudinal and reticular vascularization (Fig 5). The lateral and posterior parts of the thin-section exhibit little primary bone tissue. Laterally, the outer cortex exhibits avascular lamellar bone interrupted by woven-fibered bone with laminar and reticular vascularization (Figure D in S1 File).

**Fig 6:**
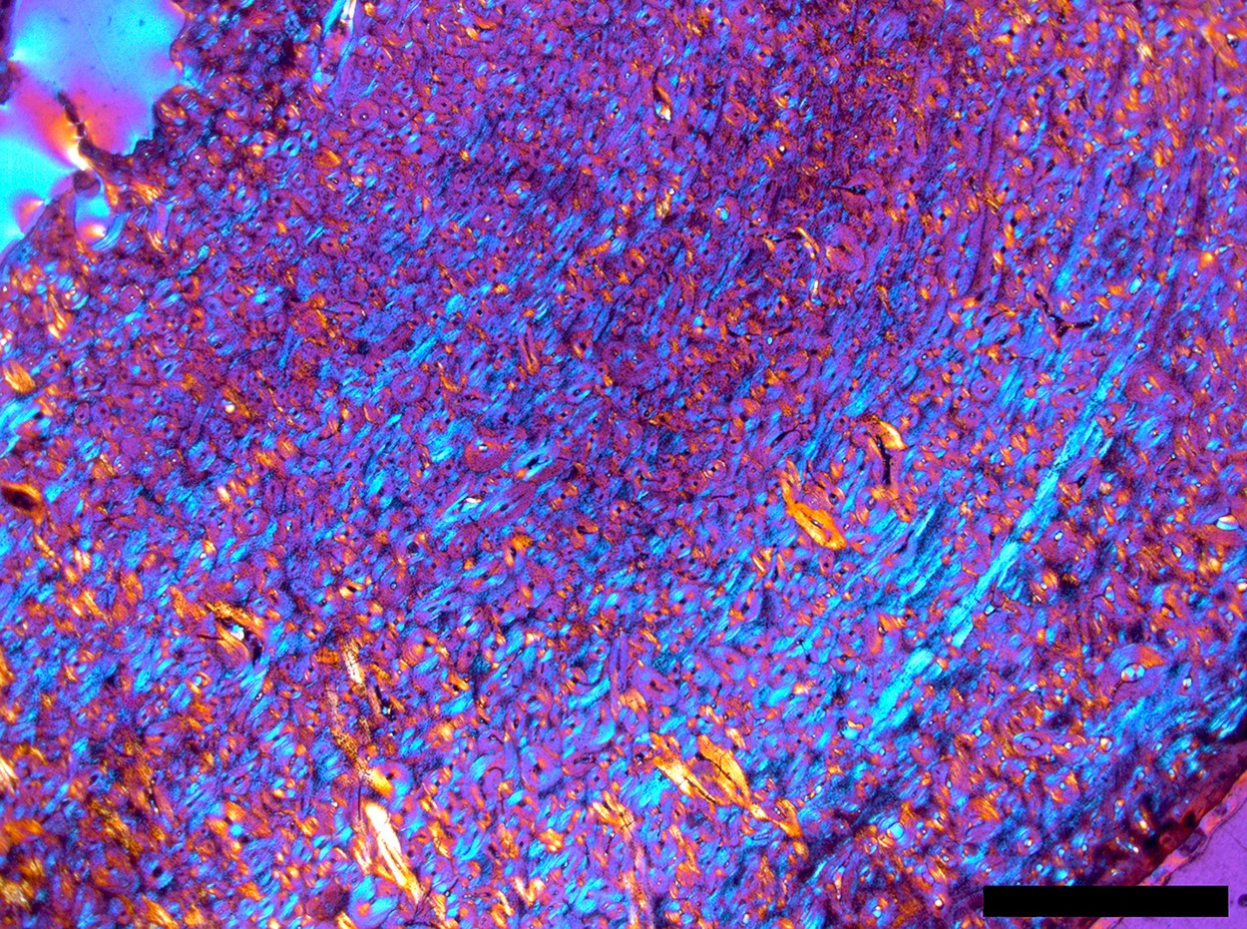
Histology of the posterior part of the femoral midshaft of *Ursus spelaeus* (MB. Ma. 10886). The thin-section is shown in cross-polarised light with lambda compensator. Note the dense Haversian tissue, comprised of several generations of secondary osteons, which extends to the outer cortex (Scale bar: 2 mm).

Bone remodelling in *U*. *spelaeus* s.l. starts in the posterior inner cortex with scattered secondary osteons. In the inner part of the cortex, especially posteriorly, dense Haversian bone is present already in young individuals. In older individuals, scattered secondary osteons are found in many parts of the cortex, but remodelling is especially prevalent anteriorly. Posteriorly, around the linea aspera—a ridge on the posterior section of femora—dense Haversian bone reaches the outer surface of the cortex (Fig 6). In their first year, the femora of *U*. *arctos* exhibit no remodelling of the bone. First signs of remodelling start in the posterior part of the bone around the linea aspera. Over time, dense Haversian bone tissue forms with several generations of secondary osteons, which reach the outer surface of the cortex. In the other regions of the bone, remodelling starts in the inner part of the cortex with scattered secondary osteons. With increasing age more scattered secondary osteons appear also in outer parts of the cortex. In old animals, a dense Haversian bone tissue forms in the inner cortex of the bone (Fig 4).

In *M*. *ursinus*, *U*. *americanus*, *U*. *deningeri*, and *U*. *maritimus* bone remodelling is also most pronounced around the linea aspera in the posterior part of the bone. In this part, Haversian bone is dense and reaches the outer part of the cortex. In other parts of the bone, especially in the inner part of the cortex, scattered secondary osteons are present. The anterior part of the femora often exhibits a higher amount of scattered secondary osteons.

### Growth marks and growth in cave bears and closely related species

**Fig 7:**
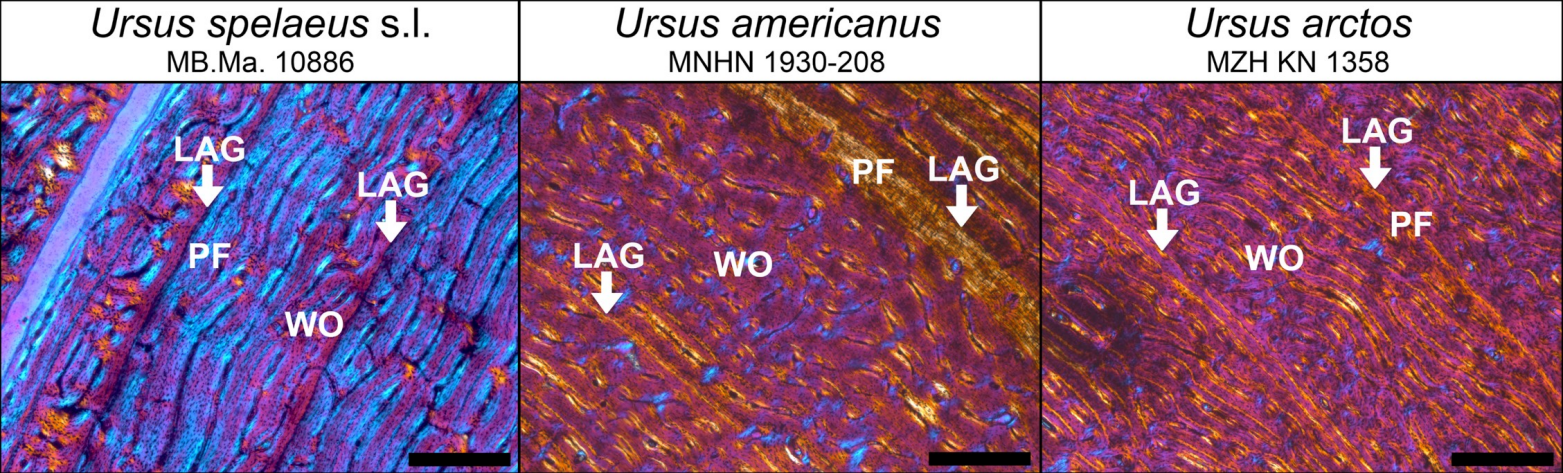
Comparison of the growth zones between *Ursus spelaeus*, *Ursus americanus*, and *Ursus arctos* (pictures under cross-polarized light with lambda compensator). White arrows indicate LAGs. Note that inner part of the growth zone exhibits more WO bone whereas the external part exhibits more PF bone. WO = woven-fibered bone, PF = parallel-fibered bone. (Scale bars: 0.5 mm).

All examined bear species and cave bear populations with preserved histology exhibit LAGs except for a few early juveniles. In cave bears, an ontogenetic series of thin-sections revealed that the first LAG is resorbed around the time the sixth one is deposited (Fig 4). The ontogenetic series of *U*. *arctos* shows that the number of LAGs is consistently below the minimum age documented for the individual, evidencing the loss of at least one LAG during ontogeny. Thus, for comparison, the loss of the first LAG and the first growth zone was inferred for all adult bear specimens within the study. Growth zones of cave bears commonly exhibit a regular pattern. After the termination by a previous LAG, the growth zone starts with the deposition of woven-fibered bone. Over time, the amount of parallel-fibered bone increases and finally the growth zone terminates with the deposition of the subsequent LAG. In contrast, in *U*. *arctos* and *U*. *americanus* the growth zone is mostly a layer of a uniform fibrolamellar complex and only close to the cessation of growth, marked by a LAG, a thin layer of parallel-fibered bone is produced, also called annulus (Fig 7). The growth zones of *H*. *malayanus*, *M*. *ursinus U*. *deningeri*, and *U*. *maritimus* are less uniform and exhibit somewhat alternating parallel-fibered and woven-fibered bone in the fibrolamellar complex (Figure J in S1 File).

The timing of the cessation of circumferential bone growth is indicated by the OCL [29]. Usually, an OCL is a layer of avascular lamellar bone potentially comprising closely spaced LAGs. The OCL of cave bears appears between LAG 9 and 13 and in *U*. *arctos* it appears between LAG 6 and 7. Skeletal maturity was reached in *U*. *americanus* after LAG 7, in *H*. *malayanus* after LAG 5, in *U*. *maritimus* after LAG 10, and in *M*. *ursinus* after LAG 6. No OCL was recorded in the available specimens of *U*. *deningeri*, suggesting that this animal did not reach skeletal maturity before the age of eleven (ten LAGs plus one resorbed, S1 Table).

**Fig 8:**
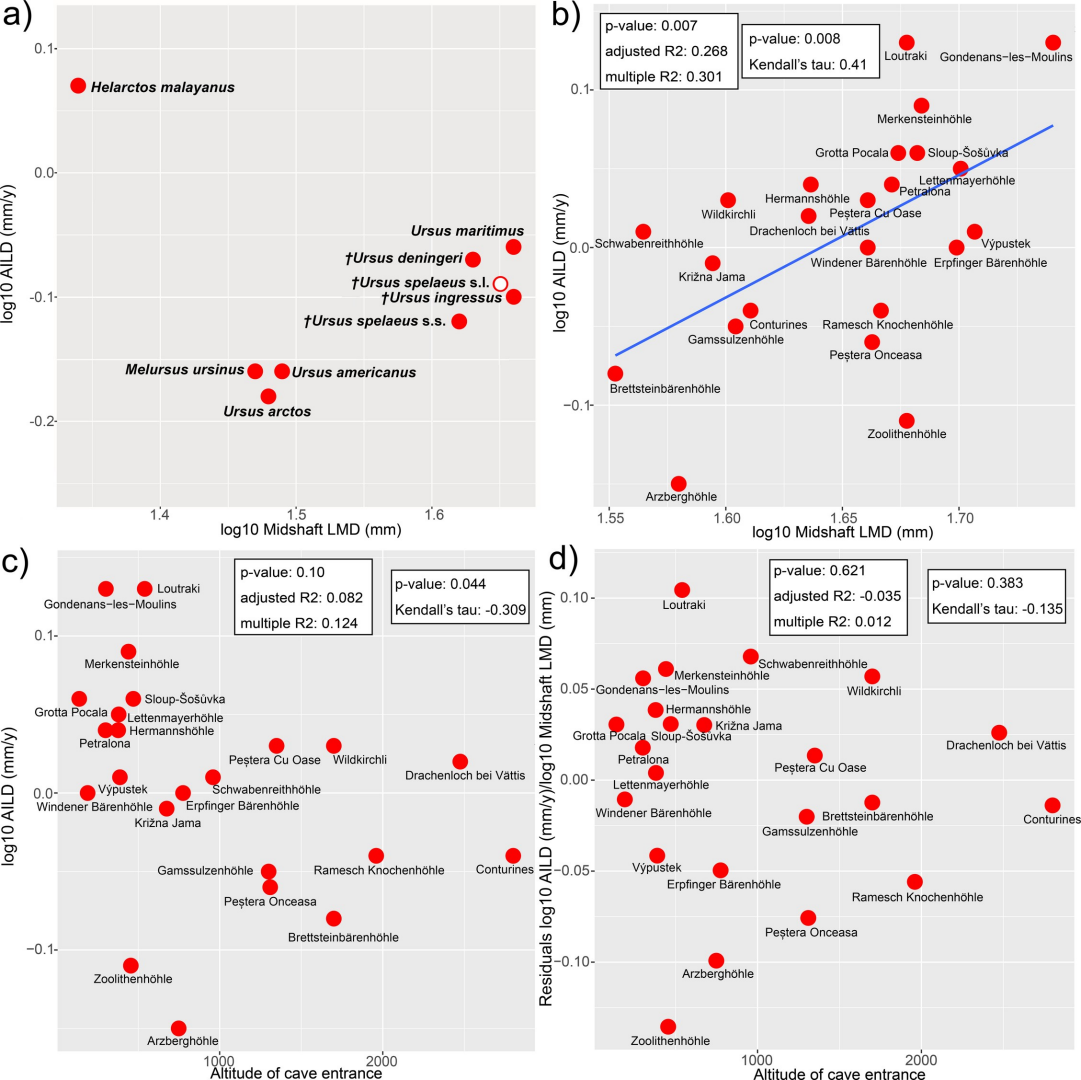
Averaged inter-LAG distance (AILD) correlation to body size and altitude. Scatter plot of: a) AILD of different bear species compared to their femoral latero-medial diameterat midshaft (LMD) as a proxy for body size (open circle not included in phylogenetic analyses), b) AILD of different cave bear localities compared to their LMD, c) AILD of different cave bear localities compared to the altitude of the locality, d) AILD/LMD-residuals compared to the altitude of the locality (results of OLS regression in the left box, results of Kendall’s tau in the right box).

The overall growth, as presented in Fig 2 and Fig 8A (using AILD as a proxy), shows that cave bears were growing in comparable speed to *U*. *maritimus*. The second best sampled species *U*. *arctos* exhibits slower growth rates. In our sampling, the fast growing species were *U*. *deningeri*, *U*. *maritimus*, and *U*. *spelaeus* s.l. (*U*. *ingressus*, *U spelaeus* s.s., the slow growing species were *U*. *americanus*, *U*. *arctos*, and *M*. *ursinus* (Fig 2, Fig 8A). For its size, according to our data, the fastest growing species was *H*. *malayanus*. However, the placement of this species in the scatterplot (Fig 8A) is unique. The phylogenetically informed analysis showed that the relationship among bear species is not significantly correlated with the scaling of their AILD (λ = 0) (Table 1, the coefficients of the OLS regression are very similar). Accordingly, mapping of AILD onto the ursid timetree (Fig 2) does not reveal clade-specific patterns.

**Table 1:** Correlation of averaged inter-LAG distance (AILD) to body size and altitude in ursids, as given by PGLS (λ = 0) and OLS regressions and Kendall’s tau analyses.

Model	Intercept	Std. Error	t	p	Slope	Std. Error	t	p	multiple R2	adjusted R2	Correlation p-value (Kendall's tau)
OLS Species: log10 Growth Rate (mm/y)/log10 Midshaft LMD (mm)	0.2361	0.4710	0.501	0.637	-0.2204	0.3068	-0.718	0.505	0.09357	-0.08772	0.3429 (0.286)
PGLS Species: log10 Growth Rate (mm/y)/log10 Midshaft LMD (mm)	0.2359	0.4709	0.501	0.638	-0.2203	0.3067	-0.7183	0.5048	0.09353	-0.08777	-
OLS Populations: log10 Growth Rate (mm/y)/log10 Midshaft LMD (mm)	-1.2761	0.4270	-2.989	0.0070	0.7777	0.2587	3.006	0.0067	0.3009	0.2676	0.0075 (0.41)
OLS Populations: log10 Growth Rate (mm/y)/Altitude	0.0377	0.0266	1.666	0.111	-3.258e-05	1.893e-05	-1.721	0.100	0.1236	0.08184	0.0441 (-0.309)
OLS Populations: log10 Midshaft LMD (mm)/Altitude	1.679	0.015	111.86	<0.0001	-3.111e-05	1.254e-05	-2.48	0.0217	0.2265	0.1897	0.0161 (-0.366)
OLS Populations: Residuals log10 log10 Growth Rate (mm/y)/log10 Midshaft LMD (mm)/Altitude	0.0089	0.0199	0.447	0.659	-8.367e-06	1.667e-05	-0.502	0.621	0.0119	-0.0352	0.3833 (-0.135)

The standardized growth among different cave bear localities showed a considerable amount of variation in AILD (Fig 8B). Within cave bears, growth was significantly correlated with size (Fig 7B, Table 1). The slowest growth was recorded for Arzberghöhle, Zoolithenhöhle, and Brettsteinbärenhöhle whereas the fastest growth was documented for Loutraki and Gondenans-les-Moulins (Fig 8B). Kendall’s tau shows that AILD was correlated with altitude of the locality (Fig 8C, Table 1). However, this correlation is due to the effect of size as shown by the absence of a correlation between AILD/midshaft LMD-residuals and altitude (Fig 8D, Table 1). Indeed, in our sample, the proxy for body size (midshaft LMD) was correlated with altitude (Table 1), thus, smaller bears are found at higher altitudes.

The comparison of the slopes and intercepts of the cave bear haplotypes *U*. *spelaeus* s.s. and *U*. *ingressus* (Table 2) revealed a significant difference in the slope of these two groups (as indicated by the significant interaction between midshaft LMD and haplotype; Table 3). The slope of *U*. *spelaeus* s.s. is slightly negative (Table 2). However, the correlation is not significant. In contrast, *U*. *ingressus* has a significant positive correlation between growth rate and latero-medial diameter of the femora, suggesting that bigger animals grew faster than smaller ones. The intercept of both groups is not significantly different (Table 3) evidencing that there is no difference in AILD between these two haplotypes.

**Table 2:** Relationship of averaged inter-LAG distance (AILD) to body size (LMD) in *Ursus spelaeus* s.s. (incl. *U*. *s*. *eremus* and *U*. *s*. *ladinicus*) and *U*. *ingressus* using OLS regressions and Kendall’s tau. Femoral latero-medial diameter at midshaft (LMD) was used as a proxy for body size.

Model	Intercept	Std. Error	t	p	Slope	Std. Error	t	p	multiple R2	adjusted R2	Correlation p-value (Kendall's tau)
*Ursus spelaeus* s.s.	0.4220	0.7282	0.5800	0.5870	-0.2797	0.4505	-0.6210	0.5618	0.0716	-0.1141	0.6486 (-0.195)
*Ursus ingressus*	-2.6615	0.6822	-3.902	0.0036	1.6225	0.4138	3.9210	**0.0035**	0.6308	0.5897	0.0014 (0.805)

**Table 3:** Influence of cave bear haplotypes on their growth. Comparison based on OLS regressions for the two different cave bear haplotypes *U*.*spelaeus* (incl. *U*. *s*. *eremus* and *U*. *s*. *ladinicus*) and *U*. *ingressus* with femoral latero-medial diameter at midshaft (LMD) as a covariate, including or excluding its interaction with the category of haplotype (upper panel and lower panel, respectively).

**Comparison of slopes *U*. *spelaeus* s.s. and *U*. *ingressus***					
log10AILD(mm/y)~log10MidshaftLMD(mm)*Haplotype	**Df**	**Sum Sq**	**Mean Sq**	**F value**	**p-value**
log10MidshaftLMD(mm)	1	0.0189	0.0189	6.900	**0.0199**
Haplotype	1	0.0017	0.0017	0.631	0.4403
log10MidshaftLMD(mm):Haplotype	1	0.0273	0.0273	9.949	**0.0070**
Residuals	14	0.0384	0.0027		
**Comparison of intercepts *U*. *spelaeus* s.s. and *U*. *ingressus***					
**log10AILD(mm/y)~log10MidshaftLMD(mm)+Haplotype**	**Df**	**Sum Sq**	**Mean Sq**	**F value**	**p-value**
log10MidshaftLMD(mm)	1	0.0189	0.0189	4.322	0.0552
Haplotype	1	0.0017	0.0017	0.395	0.5390
Residuals	15	0.0656	0.0044		

## Discussion

Using AILD as a proxy for growth, our data suggest that extant and extinct bear species grew rather uniformly, with the exception of *H*. *malayanus*. It remains unclear, if the growth documented in our sample of *H*. *malayanus* is representative for the species or not. The origin of the bone is unknown; thus, it might be possible that the animal was raised under protected conditions in a zoo. The histology itself does not show clear differences compared to the *Ursus* species, despite scattered secondary osteons. However, the amount of secondary osteons was very variable in bear species with higher sample sizes.

The observed intraspecific variation within *U*. *spelaeus* s.l. was high (Fig 8A and 8B, Table 1). The histology of *U*. *americanus* and *U*. *arctos*, however, suggests a fast growth because of the amounts of woven-fibered bone. On the other hand, *U*. *maritimus* exhibits a great amount of slowly deposited parallel-fibered and lamellar bone, which would suggest a slower growth than found based on AILD. However, in contrast to artiodactyls [18], growth periods during the year are not known for bear species. As aforementioned, bears slow down bone deposition and resorption during hibernation [26]. Thus the extent of the cessation of growth between species could be different. Additionally, the length of hibernation varies among populations of one species [50]. The observed variation in AILD among cave bears from different localities was best explained by their size, which in turn is correlated to the altitude of cave entrance. Smaller high alpine cave bear populations [13] have AILDs that are not significantly smaller than the ones from their lowland relatives. In contrast, experiments mimicking high altitude conditions have shown that growth rate is usually smaller in elevated environments due to hypoxia as well as reduced appetite [14, 51, 52]. This effect is positively correlated with altitude [14, 53]. However, findings by Elia et al. [14] suggest that this effect is not found under 1850 m altitude. Our data show that size dependent AILD drops already around 500 m above sea level, excluding the outlying caves Arzberghöhle and Zoolithenhöhle (Fig 8C).

The presence of a fibrolamellar complex in bear species follows previously described thin-sections for the genus *Ursus* [54, 55]. However, notable differences were recorded in the composition of the fibrolamellar complex. Whereas, *U*. *spelaeus* s.l., *U*. *arctos*, and *U americanus* exhibit a bone histological pattern similar to the one described by Enlow and Brown [54], the one in other bear species is characterized by higher amounts of parallel-fibered and lamellar bone. A notable difference was found in *M*. *ursinus*, with considerable amounts of longitudinal vascular canals only present in this species. One of the first histological examinations of *U*. *spelaeus* s.l., from the Drachenhöhle of Mixnitz, described lamellar bone structure within trabecular bone [56]. Later studies on cave bear histology focused on mineralogical examinations of remains to investigate taphonomic processes [57] or pathological changes to the bone [58].

The ontogenetic change of bone tissue in *U*. *arctos* and *U*. *spelaeus* s.l. mirrors findings in deer femora with the exception that remodelling in bears starts in the inner cortex [17]. The cessation of bone growth as marked by an OCL has been associated with either sexual maturity [59, 60] or skeletal maturity [16]. In *U*. *arctos*, femora are skeletally mature between 5 and 8 years [61] and animals reach sexual maturity between the age of 3.5 and 5.5 years [50]. The OCL in *U*. *arctos* is produced between 7 and 8 years (6–7 LAGs plus one resorbed) suggesting that it indicates skeletal maturity. For the other examined bear species, the appearance of the OCL cannot be linked to sexual maturity because the amount of observed LAGs usually exceeds the known age of sexual maturity [50]. In cave bears the OCL appears between LAG 9 and 13, which suggest attaining femoral skeletal maturity at an advanced age. Evidence from histological thin-sections of Cervidae also suggest that the OCL is linked to skeletal maturity [17]. The best sampled species, *U*. *spelaeus* s.l. exhibits a gradual decrease in growth rate over time. In contrast, in deer the growth rate drops after growth zone 3 and remains fairly constant afterwards [17].

The remodelling of the femora of all examined bear species generally follows a very similar spatial pattern. Haversian tissue is dense around the linea aspera and the inner part of the cortex, whereas in the rest of the bone more scattered secondary osteons are present. A similar pattern of remodelling was described by Mátyás [49]. In deer, remodelling is also strongest close to the linea aspera [17]. The linea aspera serves as point of attachments for several muscles [62]. Thus, the remodelling might be the result of increased mechanical stress in this area.

As for now, the effect of hibernation on the composition of the fibrolamellar-complex remains elusive. Hibernation is a possible explanation for the formation of zonal bone in dinosaurs [55] but mammals express zonal bone independent of hibernation [this study, 21]. Our sampling does not conclusively add further information. Although bone histology of the non-hibernating bear *M*. *ursinus* is different from the other sampled bears, the same is not true for *H*. *malayanus*, which does not hibernate either [50].

## Conclusion

Cave bear offspring had about the same size as the ones of *U*. *arctos* at birth [63–65] and thus grew faster [65] and longer (as indicated by the OCL) to attain their adult stature of 500 kg [66] or probably even up to 1,500 kg [3]. The maximum known age for cave bears is 30–32 years [67, 68]. However, most individuals did not reach that age, as mortality was often high among juveniles [67–70].

The investigated haplotypes of cave bears exhibit different growth patterns indicating variation in life history strategy during early phases of speciation. There is evidence that cave bears had a faster life history compared to close relatives because of their smaller relative brain size [71]. However, the overall pace of growth reconstructed for cave bears in this study is similar to those of their close relatives.

## Supporting information

S1 Table List of investigated specimens (with their ontogenetic stage and locality) and related histomorphometrical measurements. (XLSX)

S2 Table Averaged inter-LAG distances by species or locality for *U*. *spelaeus* s.l. (XLSX)

S1 File Additional figures depicting the observed variation in histology among different cave bear localities and the different thin-section quadrants of *H*. *malayanus*, *M*. *ursinus*, *U*. *americanus*, *U*. *arctos*, *U*. *deningeri*, and *U*. *maritimus*. (PDF)
