# Supplementary material for: Palaeohistology and life history evolution in cave bears, Ursus spelaeus sensu lato
Source: PLoS One. 2018 Nov 21;13(11):e0206791. doi: 10.1371/journal.pone.0206791 (PMC6248942; doi:10.1371/journal.pone.0206791)

Veitschegger et al. (2017) Palaeohistology and life history evolution in cave bears, *Ursus spelaeus* s.l. – Supplementary File (S1 File)

Figure A: Variation in bone histology (medial) among different cave bear localities. White arrow heads indicate LAGs and red arrow heads LAGs within the OCL. Please note that the last two red arrow heads in CuO 7528-1 and the last red arrow head in DR 35 are flipped. (Scale bars: 2 mm)

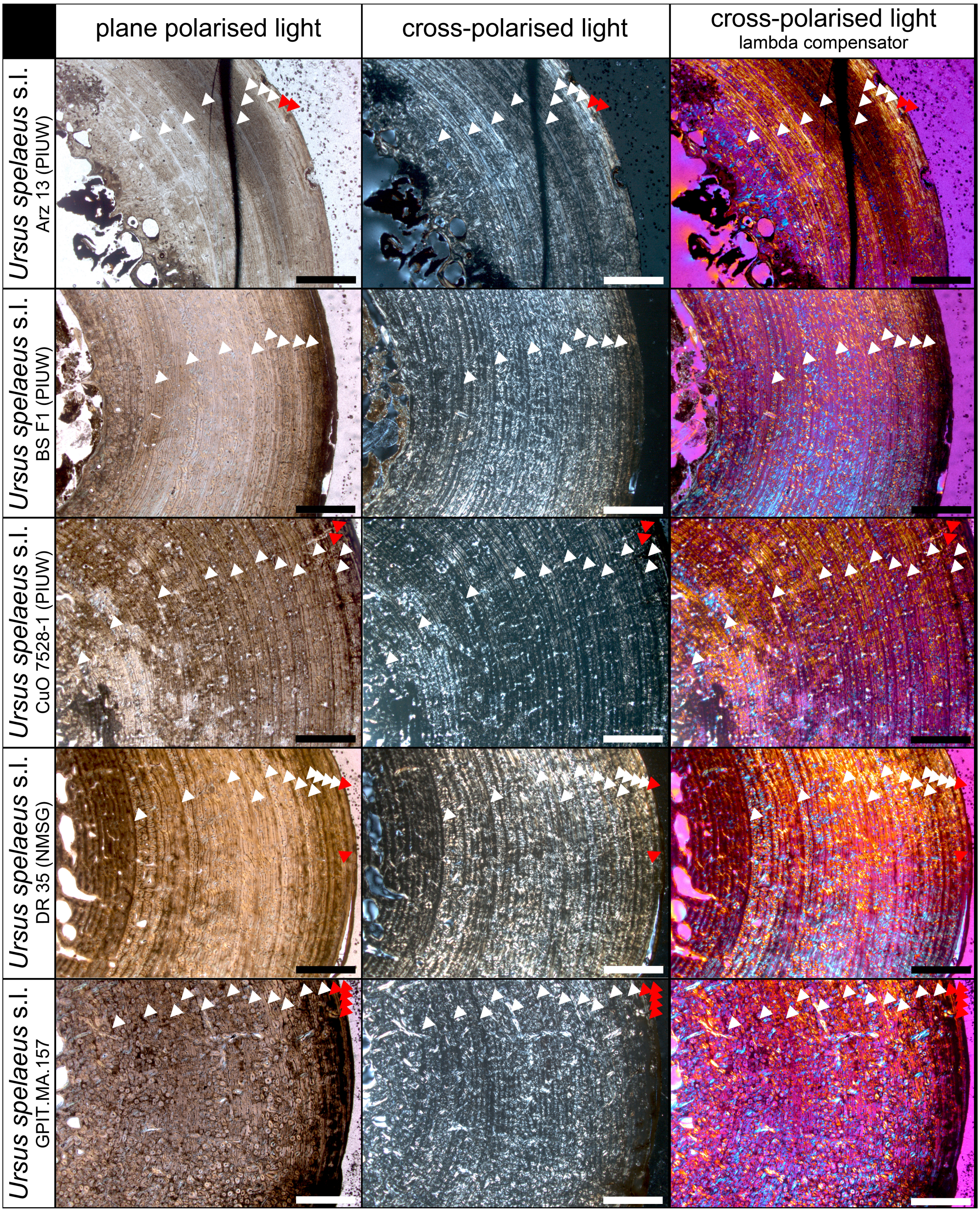

Figure B: Variation in bone histology (medial) among different cave bear localities. White arrow heads indicate LAGs and red arrow heads LAGs within the OCL. Please note that the red arrow heads of the OCL are flipped in PEC 1183 (Scale bars: 2 mm)

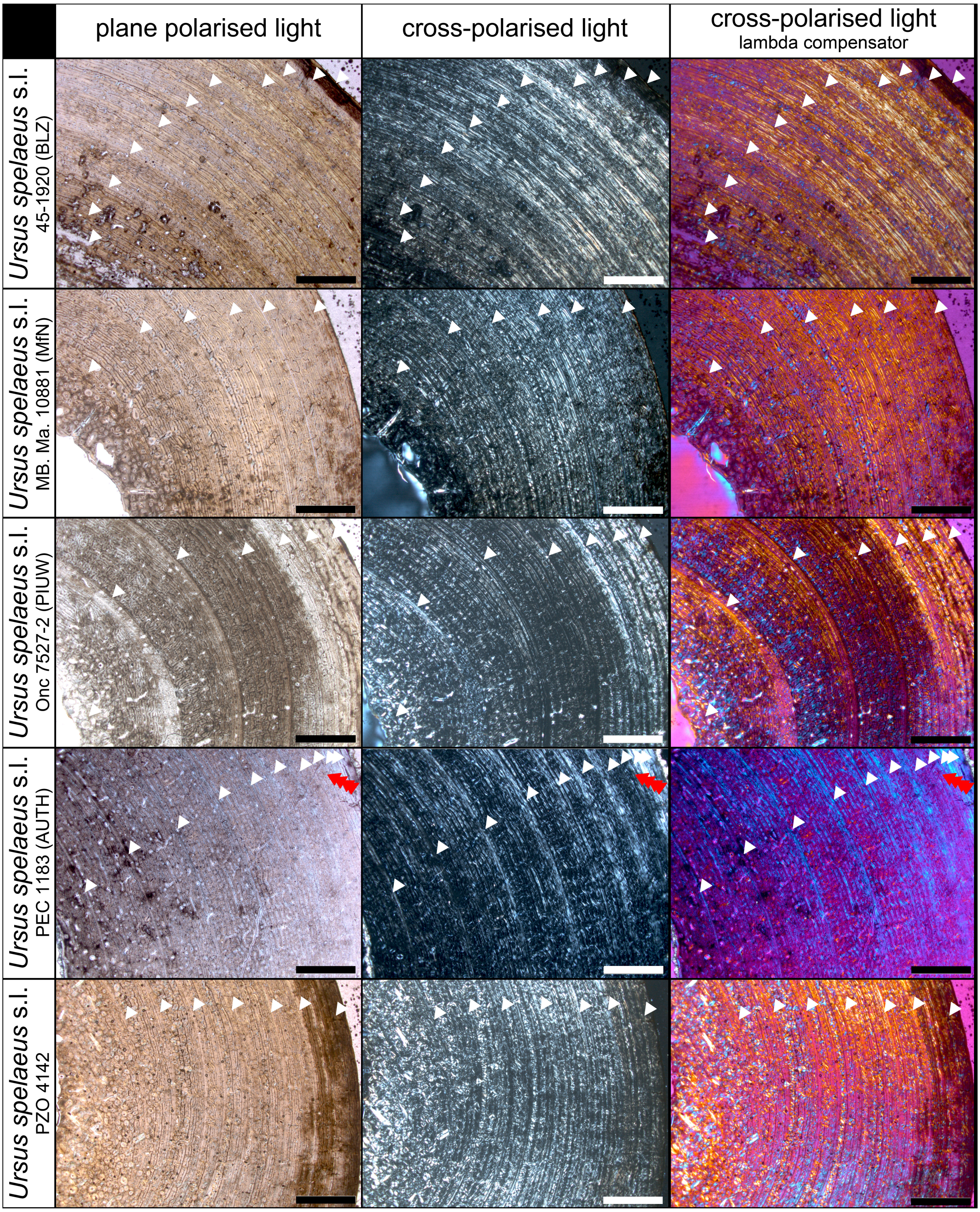

Figure C. Bone histology of the different quadrants of the midshaft of *Helarctos malayanus* femur MNHN 1914-360. (Scale bars: 2 mm)

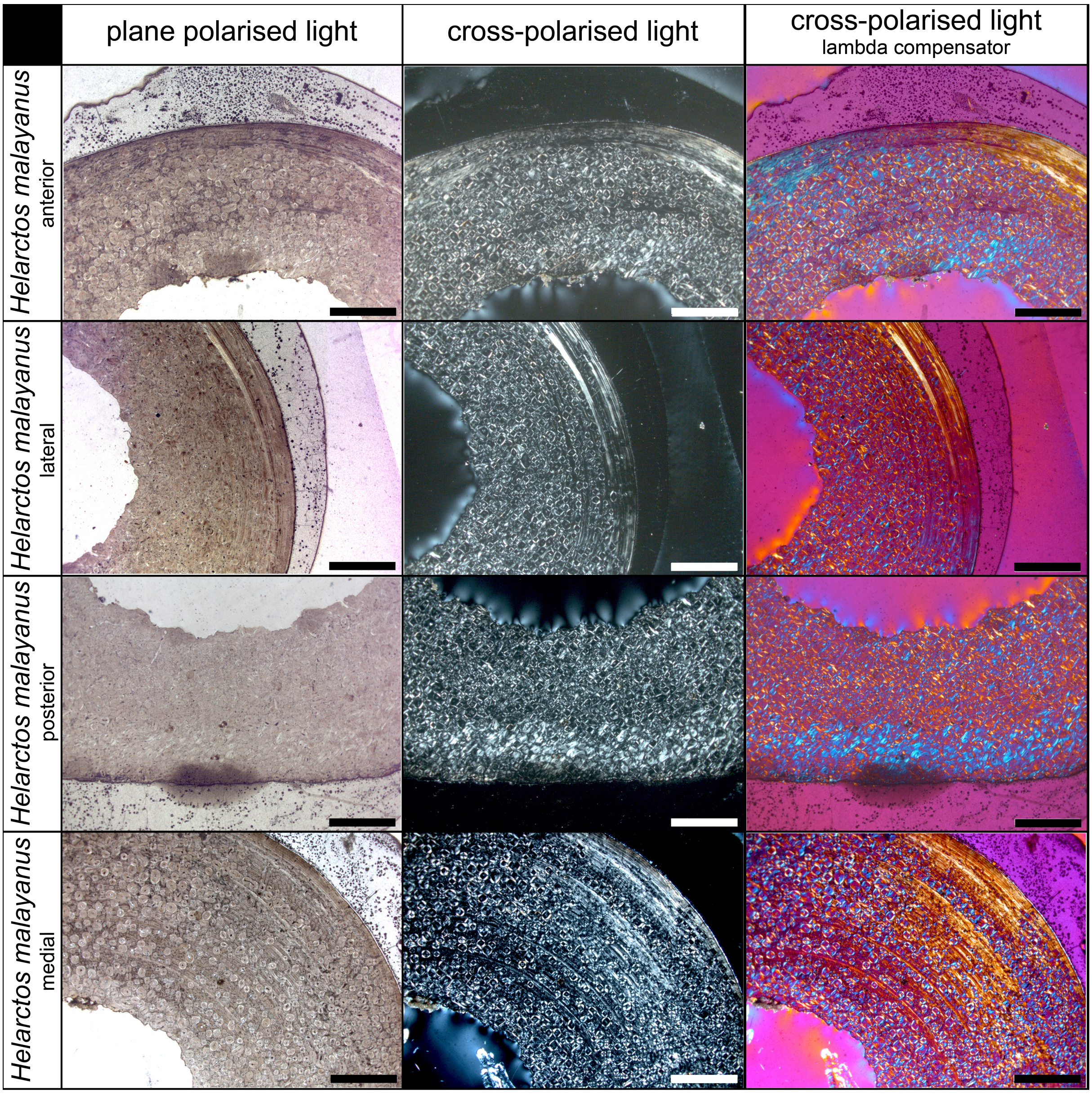

Figure D. Bone histology of the different quadrants of the midshaft of *Melursus ursinus* femur MNHN 1879-307. (Scale bars: 2 mm)

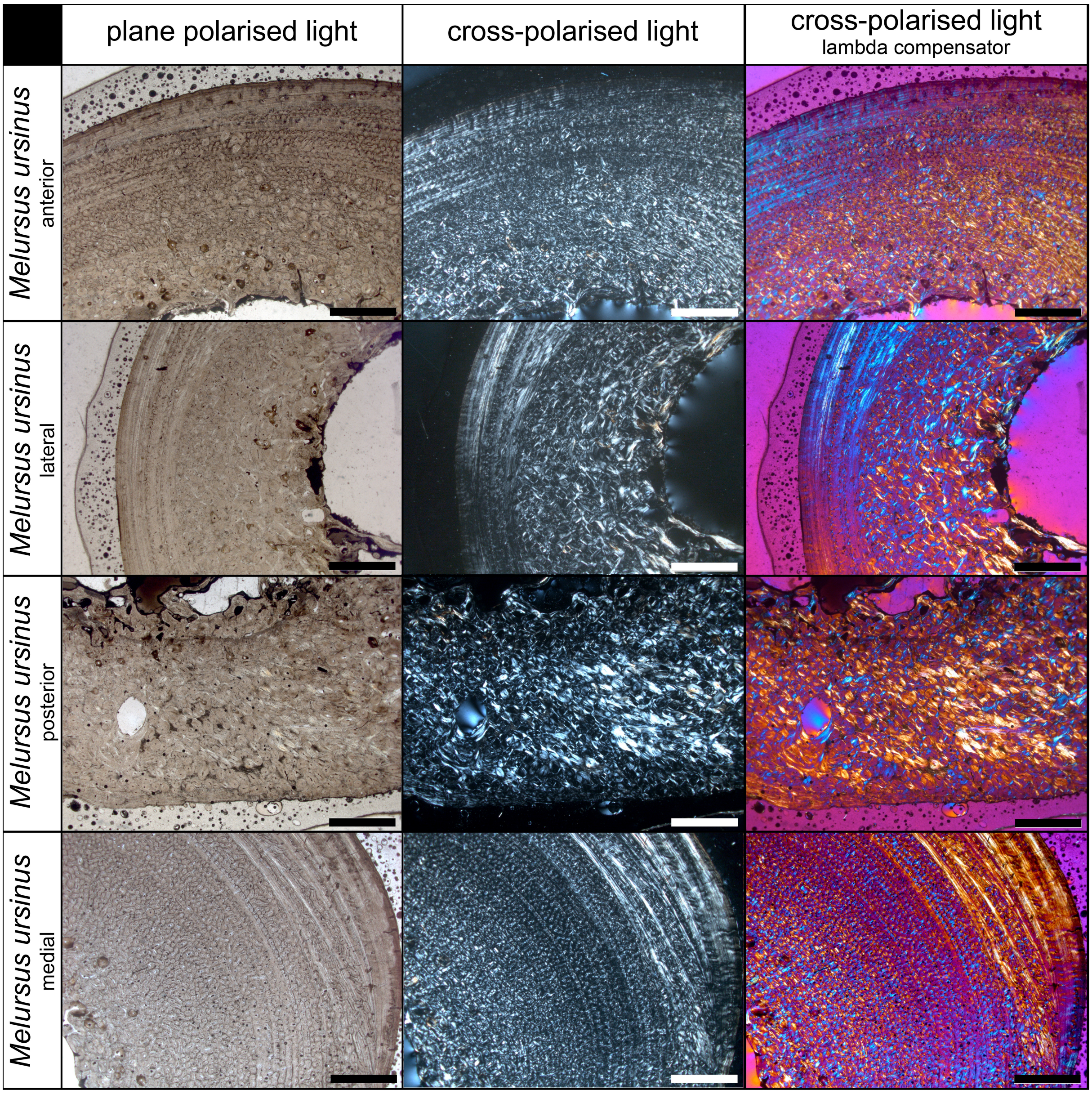

Figure E. Bone histology of the different quadrants of the midshaft of *Ursus americanus* femur MNHN 1930-208. (Scale bars: 2 mm)

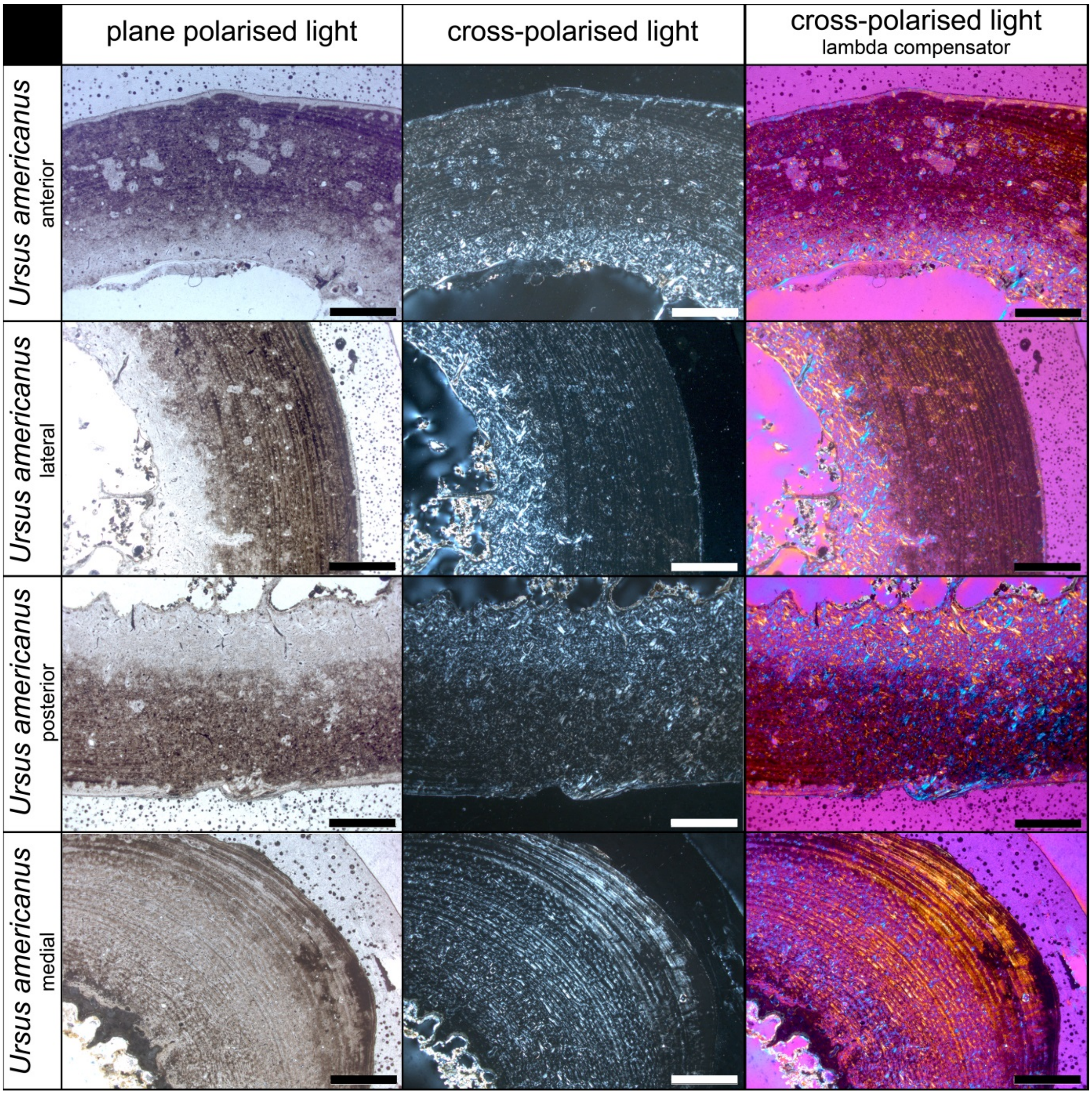

Figure F. Bone histology of the different quadrants of the midshaft of *Ursus arctos* femur MZH KN 1358. (Scale bars: 2 mm)

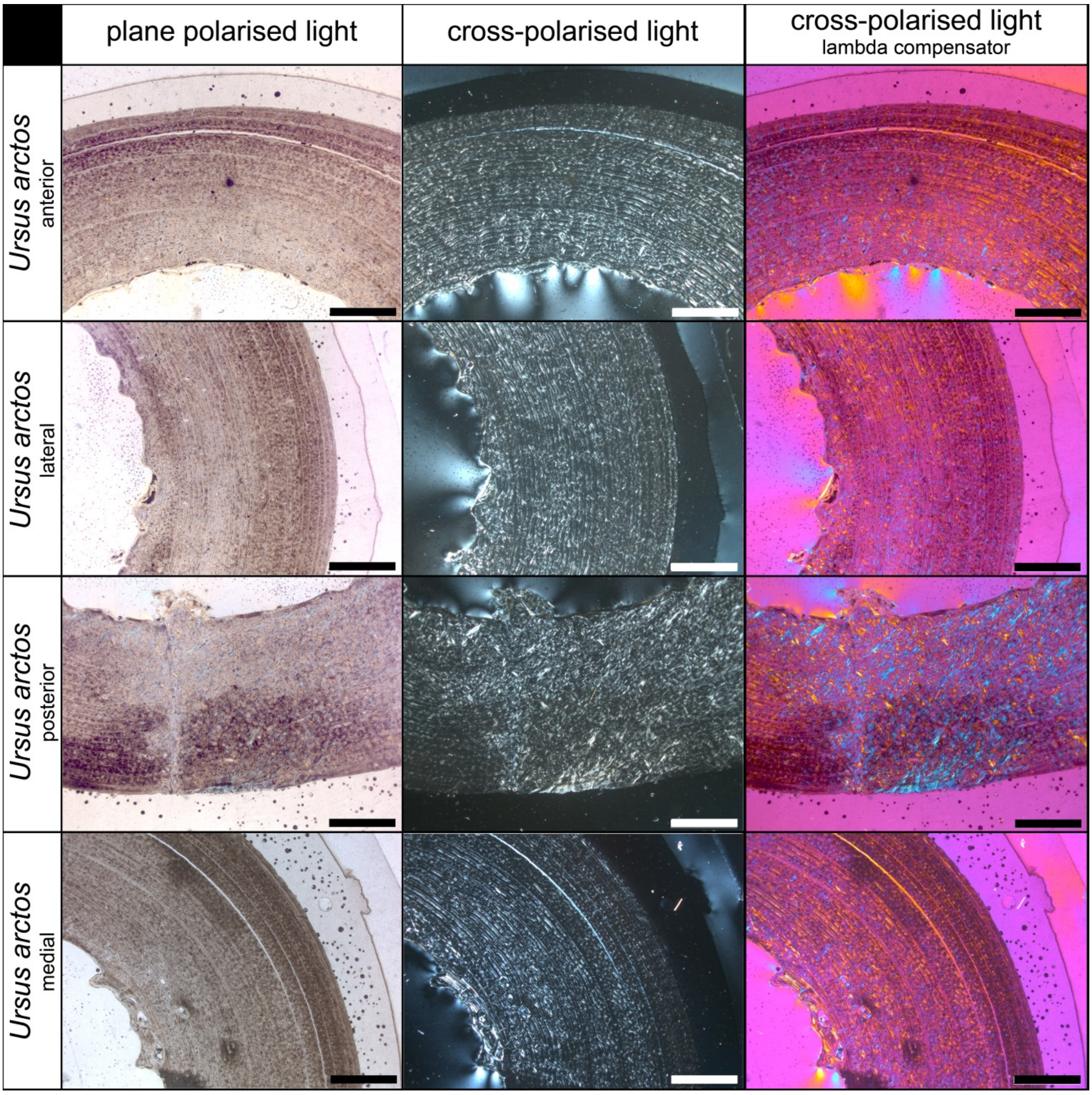

Figure G. Bone histology of the different quadrants of the midshaft of *Ursus deningeri* femur HJ 151 (PIUW). (Scale bars: 2 mm)

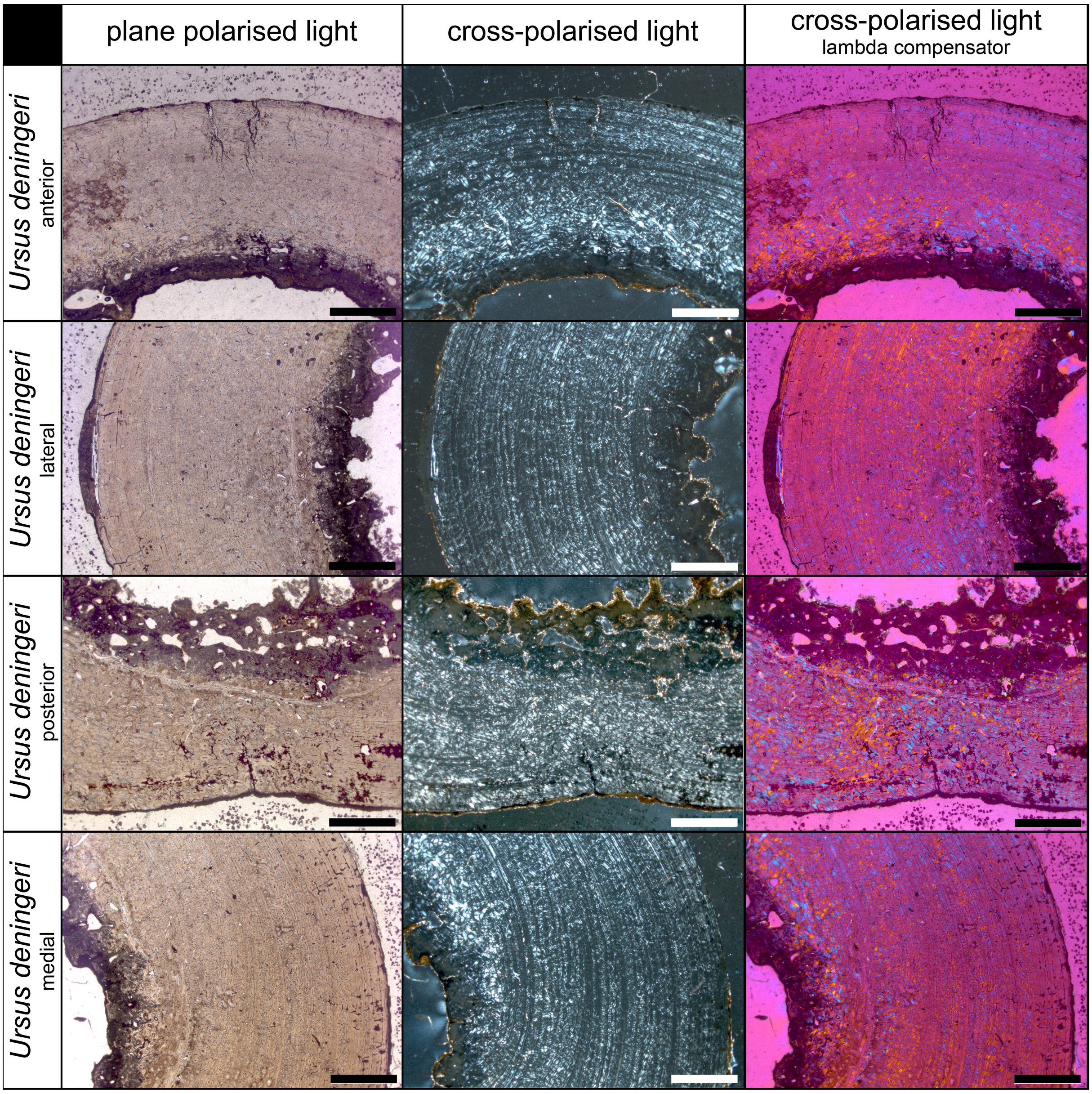

Figure H. Bone histology of the different quadrants of the midshaft of *Ursus maritimus* femur MZH UN 2355. (Scale bars: 2 mm)

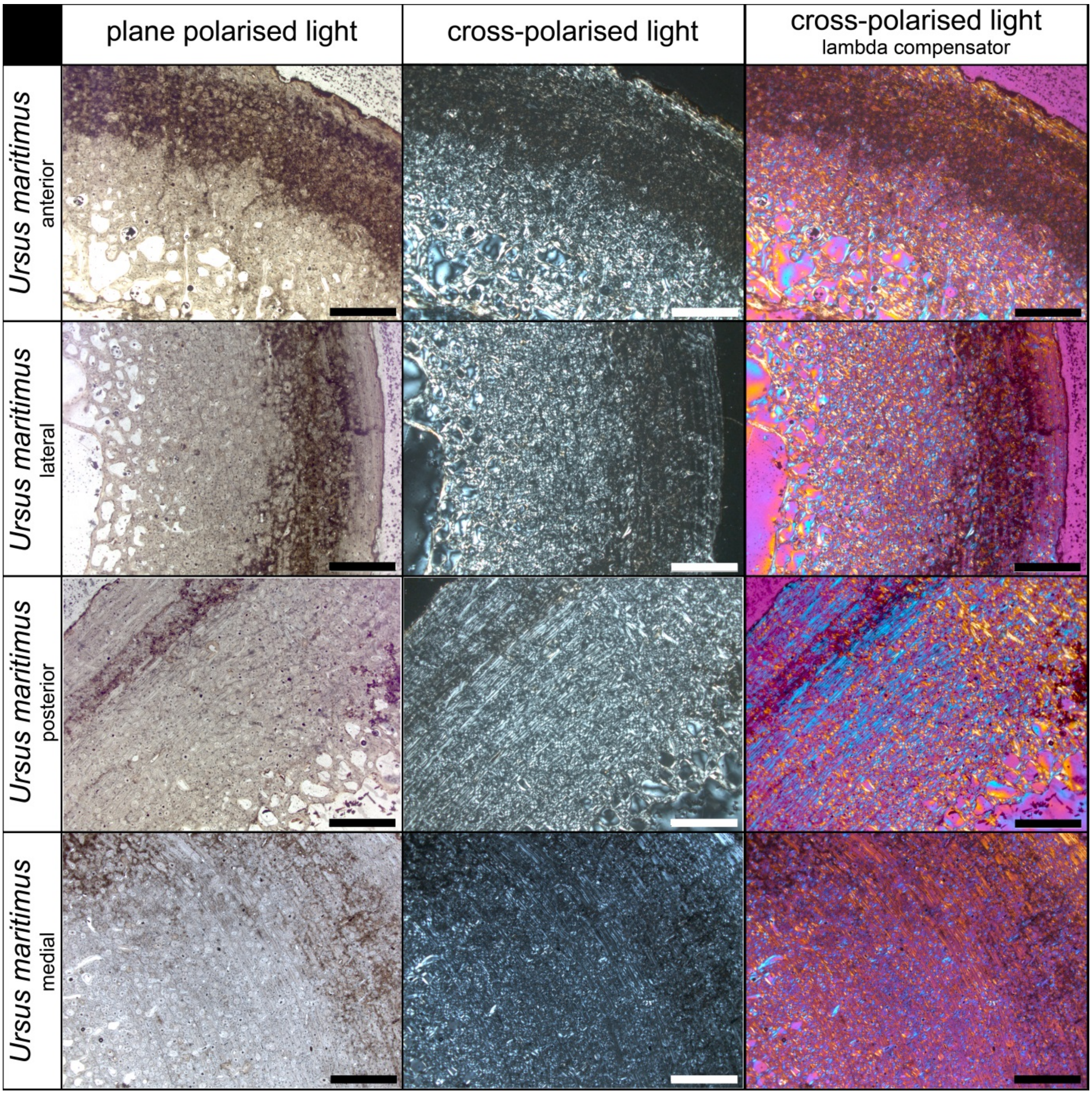

Figure I. Bone histology of the different quadrants of the midshaft of *Ursus spelaeus* femur MB. Ma. 10886. (Scale bars: 2 mm)

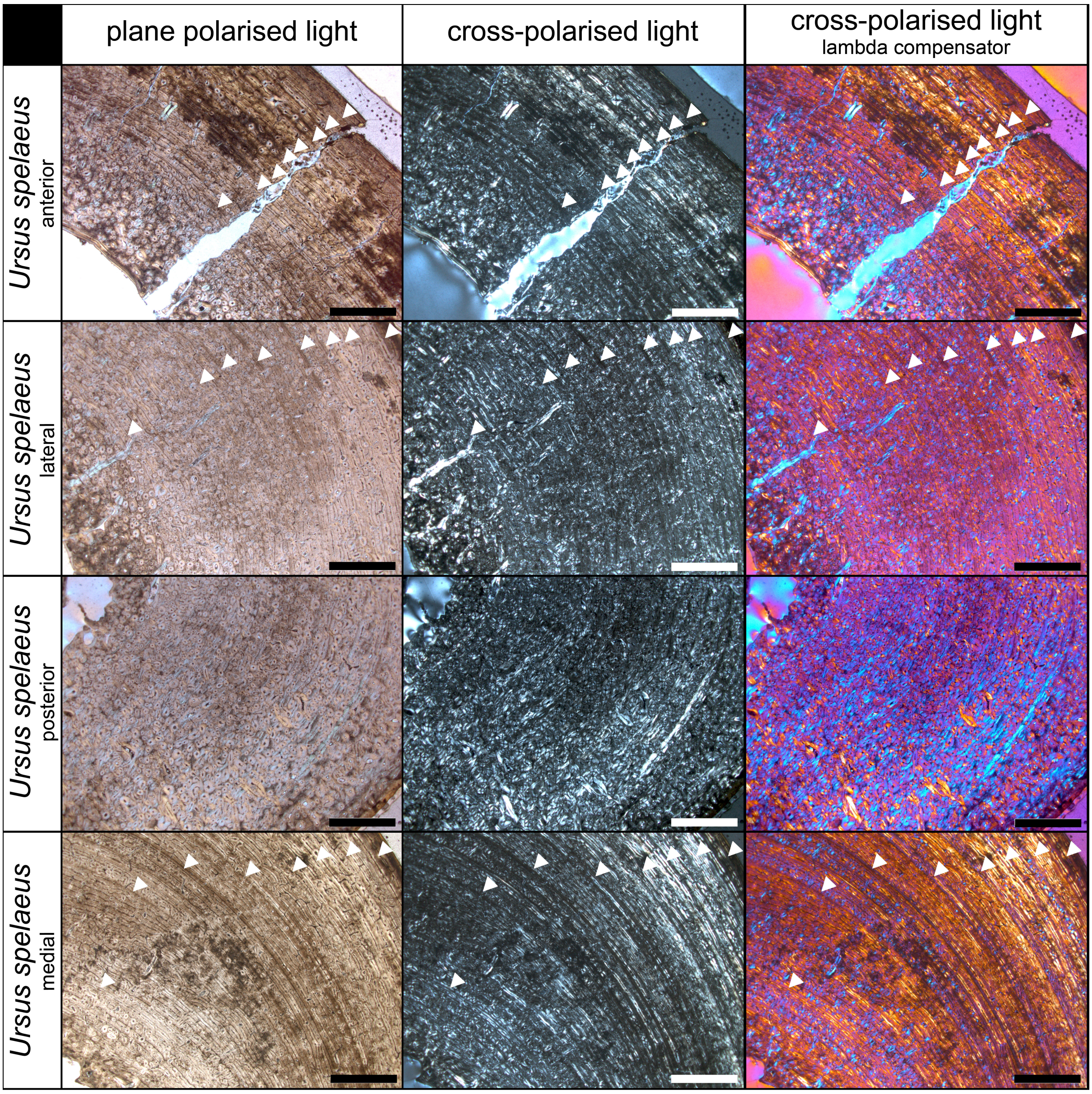

Figure J. Comparison of the growth zones among different bear species (pictures under cross-polarized light with lambda compensator). White arrow heads indicate LAGs. (Scale bars: 0.5 mm).

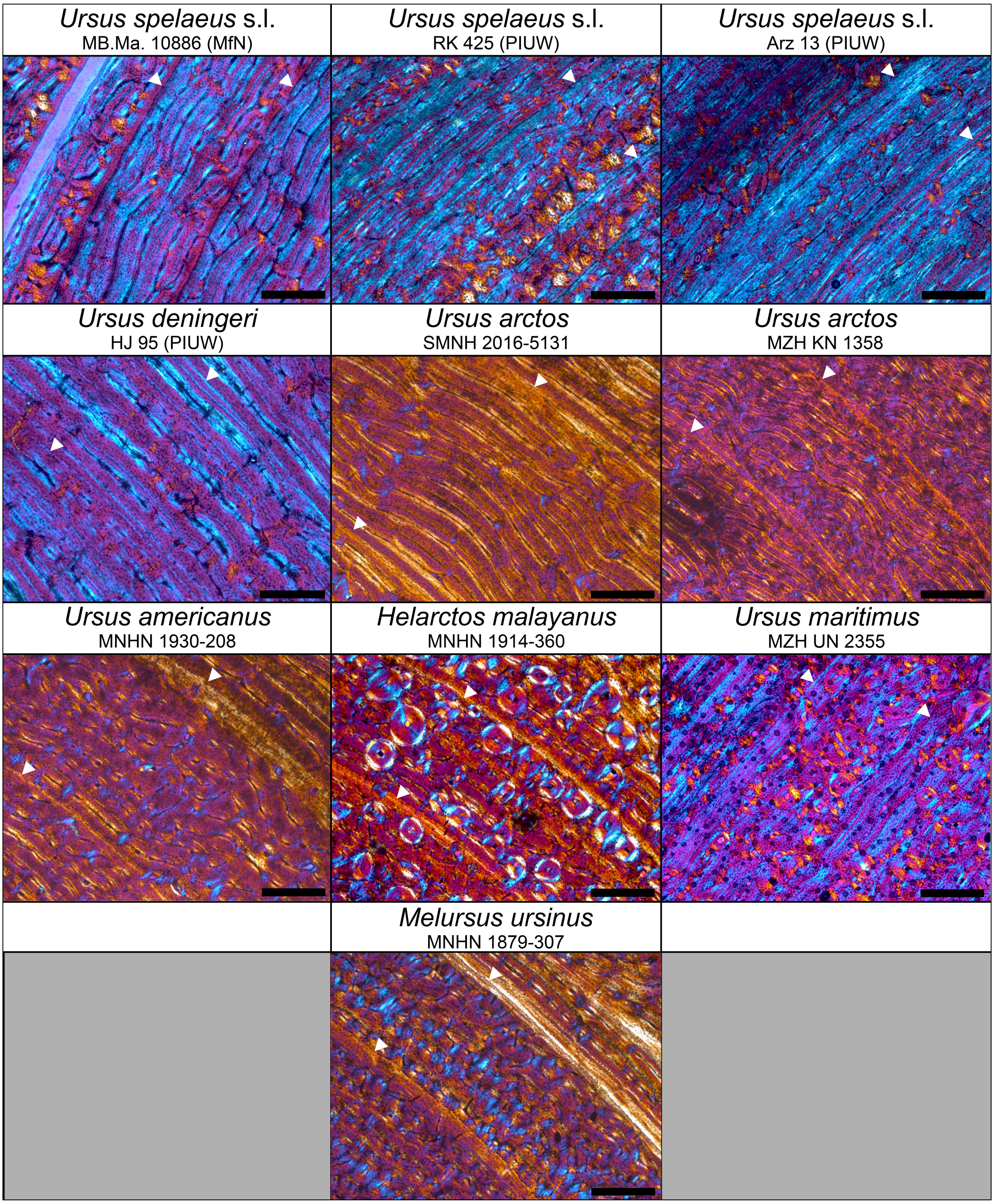

Supplement: S1 File — (PDF) [file pone.0206791.s003.pdf]
